# MyoMed205 Counteracts Titin Hyperphosphorylation and the Expression of Contraction‐Regulating Proteins in a Rat Model of HFpEF

**DOI:** 10.1002/jcsm.13843

**Published:** 2025-06-04

**Authors:** Beatrice Vahle, Antje Schauer, Antje Augstein, Maria‐Elisa Prieto Jarabo, Janet Friedrich, Peggy Barthel, Anita Männel, Norman Mangner, Siegfried Labeit, T. Scott Bowen, Axel Linke, Volker Adams

**Affiliations:** ^1^ Heart Center, University Clinic, Department of Internal Medicine, Laboratory of Molecular and Experimental Cardiology University of Technology Dresden Dresden Germany; ^2^ DZHK Partner Site Mannheim‐Heidelberg, Medical Faculty Mannheim University of Heidelberg Mannheim Germany; ^3^ Myomedix GmbH Neckargemünd Germany; ^4^ School of Biomedical Sciences, Faculty of Biological Sciences University of Leeds Leeds UK

**Keywords:** atrophy, contractile proteins, HFpEF, MuRF1, MyoMed205, sarcopenia, skeletal muscle function, titin, ZSF1

## Abstract

**Background:**

Heart failure with preserved ejection fraction (HFpEF) is associated with exercise intolerance, accompanied by alterations in the peripheral skeletal muscle (SKM). We have recently shown that titin, a giant sarcomere protein, is hyperphosphorylated in HFpEF. MuRF1 is a muscle‐specific ubiquitin E3‐ligase that interacts with titin. Blocking this interaction via small molecules (MyoMed205) can improve muscle function and mitochondrial activity in HFpEF. This study aimed to investigate the impact of MyoMed205 on titin phosphorylation and its association with changes in muscle structure and function.

**Methods:**

Obese ZSF1 rats with established HFpEF received rat chow with (*n* = 15) or without (*n* = 15) MyoMed205 and were compared with lean littermates (*n* = 15), serving as controls. After 12 weeks, in vitro SKM force, atrophy and titin—as well as contractile protein expression—were evaluated (soleus and extensor digitorum longus [EDL]). Statistical analysis was performed via multiple unpaired *t*‐test or one‐way ANOVA.

**Results:**

In HFpEF, titin hyperphosphorylation by 13% in the EDL (*p* = 0.09) and 14% (*p* = 0.03) in the soleus muscle was evident. This hyperphosphorylation was driven in part by an increase in S11878 phosphorylation (EDL: +68%, *p* = 0.004; Sol: +23.8%, *p* = 0.03), which was linked to myofiber atrophy (*r* = −0.68, *p* = 0.006) and a decline in maximal specific muscle force (*r* = −0.54, *p* = 0.008). In the EDL, significant changes in protein expression related to atrophy (MuRF1 [+24.9%, *p* = 0.02], GDF8 [+20.6%, *p* = 0.09]) and calcium handling (slow troponin C [−46%, *p* = 0.02], fast troponin I [+35.8%, *p* = 0.02]) were found in HFpEF. All of the above‐mentioned effects in HFpEF were almost completely abolished by MyoMed205 treatment, and significantly elevated titin expression was visible (+19.7%, *p*_con_ = 0.04, *p*_HFpEF_ = 0.01).

**Conclusions:**

Titin hyperphosphorylation may negatively impact skeletal muscle integrity and function in HFpEF. MyoMed205 reduced titin hyperphosphorylation and was associated with preserved skeletal muscle function and mass. Further studies are necessary to confirm the direct role of titin hyperphosphorylation on muscle function and to evaluate the therapeutic potential of MyoMed205 in HFpEF.

**Abbreviations**
con: control
EDL: extensor digitorum longus
FHL1: four and a half LIM domain 1
GDF8: myostatin
HF: heart failure
HFpEF: heart failure with preserved ejection fraction
LVEDP: left ventricular end‐diastolic pressure
LVEF: left ventricular ejection fraction
MAFbx: muscle atrophy F‐box
MHC: myosin heavy chain
MLC: myosin light chain
MuRF1: muscle RING‐finger protein 1
PVDF: polyvinylidene fluoride
S11878: Serin11878
SKM: skeletal muscle
Sol: soleus
Tm: tropomyosin
TnC: troponin C
TnI: troponin I
TnT: troponin T
UbiK48: lysine 48 ubiquitin

## 1 Background

Approximately 50% of all heart failure (HF) patients have a preserved left ventricular ejection fraction (LVEF) but suffer from diastolic dysfunction and are classified as HF patients with preserved ejection fraction (HFpEF) [1, 2, 3]. Exercise intolerance is a hallmark in HF, irrespective of aetiology, and adversely affects both quality of life and prognosis [4]. Structural changes in skeletal muscle (SKM) [5, 6] including a fibre‐type switch [7, 8], fibre atrophy [5], mitochondrial dysfunction [9] and reduced blood flow and capillarity [10, 11, 12, 13] have been shown to partially contribute to exercise intolerance. In addition, changes in contractile proteins such as titin and myosin heavy chain (MHC) are thought to play a role too [14, 15].

With respect to sarcomere function, titin represents the largest protein known and is essential for the stability of the sarcomere as a contractile unit and its assembly, as well as the maintenance of sarcomere proteins [16, 17]. In HFpEF, titin hypophosphorylation is a well‐studied regulatory mechanism for myocardial stiffness [18]. In contrast to the myocardium, we recently reported a hyperphosphorylation of titin in the peripheral SKM tissue of an HFpEF animal model [14]. This hyperphosphorylation was accompanied by reduced muscle force, atrophy and dysregulation of Z‐disc–related proteins. The conjunction of titin hyperphosphorylation and reduced muscle performance is further supported by the observation that exercise training, which is known to attenuate physical limitations in HFpEF [1, 2, 19], reduced titin hyperphosphorylation in an experimental rat model of HFpEF [14]. Therefore, the modulation of titin phosphorylation may offer a novel therapeutic opportunity to influence SKM function and exercise performance in HFpEF.

A recent study in cardiomyocytes proposed that some muscle‐specific ubiquitin E3‐ligases ubiquitinate titin domains for targeted degradation by either autophagy or ubiquitin–proteasome systems [20]. Muscle RING‐finger protein 1 (MuRF1) is a ubiquitin E3‐ligase that is expressed primarily in striated muscle and the myocardium [21] and its expression is associated with the progression of muscle atrophy and SKM dysfunction in various pathological conditions [22].

With respect to potential MuRF1 targets, a yeast two‐hybrid screen of SKM cDNA libraries with MURF1 baits identified eight myofibrillar proteins, including titin [23]. Therefore, modulating the binding of MuRF1 to titin may have consequences with respect to muscle function and titin degradation. MyoMed205, identified by screening a chemical library for molecules interfering with the binding of MuRF1 to titin [24], was able to modulate SKM atrophy and performance in different diseases, including models of HFrEF [19], cardiac cachexia [24] and tumour‐associated muscle atrophy [25]. In the context of titin modulation, feeding MyoMed205 to HFpEF rats resulted in increased phosphorylation of titin in the myocardium compared with that in untreated HFpEF animals [5].

Given that HFpEF is associated with titin hyperphosphorylation in the SKM and that MyoMed205 can regulate titin phosphorylation in the myocardium, we aimed to determine the impact of MyoMed205 on titin phosphorylation and its secondary modifications in the SKM of an experimental rat model of HFpEF. We further evaluated changes in the expression of key contractile proteins that may be associated with any positive effects. In summary, our studies show that MyoMed205 has a positive impact on muscle function and the expression of contraction‐regulating proteins and leads to a reduction of titin hyperphosphorylation in the skeletal muscle of HFpEF.

## 2 Materials and Methods

### 2.1 Animals

Female ZSF1‐lean (*n* = 15) (control) and ‐obese (*n* = 30) (HFpEF) rats (Charles River, Sulzfeld, Germany) were included in the present study. At the age of 20 weeks, a randomized group of 15 ZSF1‐obese rats received rat chow that contained MyoMed205 (0.1% w/w) (treatment group, MyoMed205), whereas the remaining 15 ZSF1‐obese rats received normal rat chow (placebo group, HFpEF). After 12 weeks of treatment, the animals were sacrificed to assess the muscle function of the soleus (Sol) as a slow SKM and the extensor digitorum longus (EDL) as a fast SKM. Additionally, muscle tissue (Sol and EDL) was snap frozen and stored at −80°C or fixed with 4% PBS‐buffered formalin and embedded in paraffin. A detailed description of the study design and animal characteristics can be found in a recently published study from our group [5].

Furthermore, 30 ZSF1‐lean and 30 ZSF1‐obese animals were purchased and randomly selected at the ages of 6, 10, 15 (five ZSF1‐lean and five ZSF1 obese animals at each time point) and 20 weeks (10 ZSF1‐lean and 10 ZSF1‐obese animals). The animals were sacrificed, and functional analyses on the EDL and the Sol were performed [26].

### 2.2 Skeletal Muscle Function

Muscle function was assessed as recently described [5]. Briefly, EDL and Sol were dissected and vertically mounted in an organ bath, filled with Krebs–Henseleit buffer (1205A: Isolated Muscle System—Rat, Aurora Scientific Inc., Ontario, Canada). Muscle function was assessed ex vivo, via platinum electrodes that stimulated the muscle with a supra‐maximal current (700 mA, 500‐ms train duration, 0.25‐ms pulse width) from a high‐power bipolar stimulator (701C; Aurora Scientific Inc., Ontario, Canada). The muscle was restrained to an optimal length (Lo) defined by maximal produced twitch force. To measure maximal performance, a force‐frequency protocol was performed at 1, 15, 30, 50, 80, 120 and 150 Hz, separated by 1‐min rest intervals.

### 2.3 Analysis of Titin

Titin expression and its phosphorylation status were monitored by vertical gel agarose gel electrophoresis (VAGE). Therefore, snap frozen tissue was pulverized and dissolved in urea buffer (8‐mol/L urea, 2‐mol/L thiourea, 0.05‐mol/L Tris pH 6.8, 0.075‐mol/L DTT, 3% SDS), containing a protease‐ and phosphatase‐inhibitor mixture (Serva, Heidelberg, Germany), at a ratio of 1:50 (weight/volume). The tubes were carefully inverted several times and heated for 10 min at 60°C to fully solubilize the tissue. After a quick centrifugation (30 s at 4.000 × g), a small aliquot of the supernatant was removed for protein quantification (660‐nm protein assay, Thermo Fischer Scientific, Waltham, MA, USA). Thereafter, glycerol, containing traces of bromophenol blue, was added (final concentration 25%), followed by a centrifugation step (10 min at 13.200 × g). The supernatant was collected, and the samples were aliquoted and stored at −80°C until analysis. For running the gels, 4–8 μg of protein was loaded on 1% agarose gels as described by Zhu and Guo [27], and a current of 15 mA was applied for 5 h. To assess the amount of phosphorylated titin and MHC, the gels were stained with Pro‐Q Diamond Phosphoprotein Gel Stain. Afterwards, a Sypro Ruby gel stain was used to determine protein expression (titin, MHC and nebulin) (both Thermo Fischer Scientific, Waltham, MA, USA) according to the manufacturer's recommendation. For the assessment of protein expression, densitometry was performed using the 1D scan software package version 15.08b (Scanalytics Inc., Rockville, MD, USA). Measurements for total titin and nebulin were normalized to MHC expression, whereas phosphorylation was assessed through the ratio to unmodified protein.

### 2.4 Histological Analyses

To quantify SKM atrophy, myofiber cross‐sectional area (CSA) was measured. The paraffin‐embedded Sol and EDL tissues were sectioned (4 μm), mounted on glass slides and stained with haematoxylin and eosin. The CSA of the fibres was evaluated through imaging software (Zen imaging software, Zeiss, Jena, Germany). A minimum of 100 fibres in at least five randomly chosen areas were measured per muscle.

### 2.5 Western Blot Analysis

For normal SDS‐PAGE, frozen SKM tissue was homogenized in RIPA buffer (50‐mmol/L Tris pH 7.4, 1% NP‐40, 0.25% Na‐deoxycholate, 150‐mmol/L NaCl, 1‐mmol/L EDTA) supplemented with a protease‐inhibitor mixture (Inhibitor Mix M, Serva, Heidelberg, Germany). The protein concentration was assessed (BCA assay, Pierce, Bonn, Germany), and 5–20 μg of protein was loaded onto a gel.

All proteins were transferred to PVDF membranes with a semi‐dry blotter (VWR). The membranes were incubated with the following primary antibodies overnight at 4°C: myosin light chain (MLC) 1 (15814‐1‐AP), SMYD2 (21290‐1‐AP, 1:2000), α‐actin (23660‐1‐AP), α‐actinin (14221‐1‐AP, 1:2000), MYOT (10731‐1‐AP, 1:2000), FHL1 (10991‐1‐AP, 1:1000), GDF‐8 (19142‐1‐AP, 1:1000), tropomyosin (11038‐1‐AP, 1:2000), troponin C (13504‐1‐AP, 1:1000), troponin T (19729‐1‐AP, 1:1000) (all Proteintech, Planegg‐Martinsried, Germany), troponin I (ab184554, 1:2000), telethonin (ab184554, 1:1000), MAFbx (ab168372, 1:1000), UbiK48 (ab140601, 1:1000) (all Abcam, Cambridge, UK), MuRF1 (Santa Cruz, Dallas, TX, USA, sc398608, 1:200), titin N2A (Myomedix, Neckargemünd, Germany, 1 μg/mL) and ph‐S11878 (generously provided by Prof. Dr. W. Linke, University Münster, 1:1000). The next day, the membranes were incubated with a horseradish peroxidase‐conjugated secondary antibody, and the protein signal was visualized via enzymatic chemiluminescence (Super Signal West Pico, Thermo Fisher Scientific Inc., Bonn, Germany). Densitometry was used for quantification (1D scan software package version 15.08b; Scanalytics Inc., Rockville, MD, USA). Measurements were normalized to the loading control GAPDH (1:10000; HyTest Ltd., Turku, Finland). For the measurement of ubiquitinated MHC, samples were separated on a 1% agarose gel and transferred to a PVDF membrane. The membrane was stained with an anti‐UbiK48 antibody, and the specific band at 250 kDa representing ubiquitinylated‐MHC was quantified. Total MHC was assessed by staining the PVDF membrane with Ponceau S, and the ratio ubi‐MHC/total MHC was calculated. MLC‐2 expression and phosphorylation were assessed as recently described [28, 29, 30]. Secondary modifications were calculated as the ratio of modified to unmodified protein, and MLC‐2 expression was normalized to the total protein amount. The data are presented as x‐fold change, relative to control.

### 2.6 Statistical Analyses

Statistical analysis was performed via multiple unpaired *t*‐test or one‐way ANOVA followed by Tukey's post hoc test (for parametric data) or the Kruskal–Wallis test followed by Dunn's post hoc test (for nonparametric data). The values are reported as mean ± standard error of the mean (SEM), and *p* < 0.05 was considered significant. GraphPad Prism 9.4.0 was used for statistical analysis.

## 3 Results

### 3.1 Animal Characteristics

A detailed characteristic of the animals can be found in earlier studies of our group [5, 26]. Briefly, at 6 weeks of age, we detected diabetes, hypertension, obesity and hyperlipidaemia in the ZFS1 rats. Beginning HFpEF was detected in ZSF1 rats from 10 weeks onwards as determined by echocardiographic and invasive measurements. Myocardial BNP increased continuously over time, reaching statistical significance at 20 weeks of age. Furthermore, at an age of 20 weeks, ZSF1 rats showed a preserved left ventricle ejection fraction, increased E/é and elevated left ventricular end‐diastolic pressure (LVEDP) and NT‐proBNP levels.

At the age of 20 weeks, rats showed all signs of HFpEF, and hence, treatment with MyoMed205 was started at this age. After 12 weeks, myocardial stiffness, LVEDP, E/é and NT‐proBNP levels were improved in MyoMed205‐treated HFpEF rats as compared with untreated ZSF1 rats [26].

### 3.2 Time‐Dependent Alterations in SKM During HFpEF Progression (Figure 1)

**FIGURE 1:**
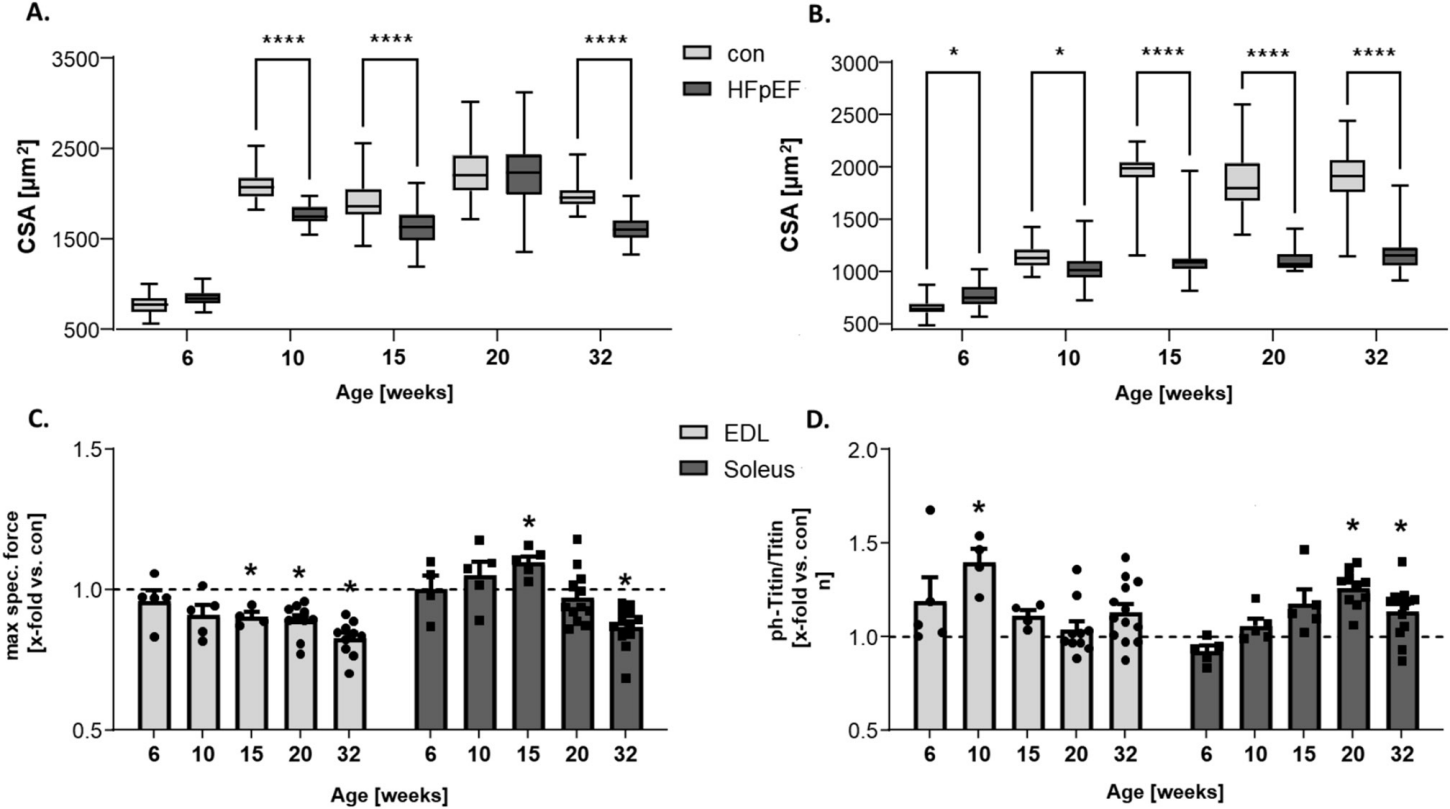
SKM rearrangement occurs earlier in the EDL than in the Sol during HFpEF development. (A) CSA soleus, (B) CSA EDL, as well as (C) maximal specific force and (D) titin phosphorylation of soleus and EDL from ZSF1‐control (con) (represented as scattered line) and ZSF1‐HFpEF rats (HFpEF). Samples were obtained from 6‐, 10‐, 15‐, 20‐ and 32‐week‐old rats. The results are expressed as median ± Min–Max (*n* = 4–5 per group) **** *p* < 0.0001 (A, B) or as x‐fold change versus control (scattered line, set to 1.0) ± SEM (*n* = 4–12 per group) * *p* < 0.05 versus con (C, D).

To provide a comprehensive temporal understanding of progressive SKM changes in response to HFpEF development, we measured muscle atrophy, contractile function and titin phosphorylation in 6‐, 10‐, 15‐, 20‐ and 32‐week‐old HFpEF rats and compared them to lean littermates (control). The Sol, as well as the EDL, showed early signs of atrophy (Figure 1A,B) from 10 weeks on. With respect to specific muscle force (Figure 1C), a significant reduction in the EDL was already visible at 15 weeks of age. The Sol in contrast showed a reduced maximal specific force, only at 32 weeks of age. Additionally, a significant hyperphosphorylation of titin was already evident in the EDL of 10‐week‐old animals but became less pronounced in older animals (Figure 1C). Interestingly, we observed titin hyperphosphorylation in the Sol already in 20‐week‐old ZSF1‐HFpEF rats that were maintained until 32 weeks (Figure 1D). Detailed force–frequency curves for both muscles in these groups have been previously published by our group [26].

### 3.3 Effects of MyoMed205 in HFpEF on SKM Atrophy and Force (Figure 2)

**FIGURE 2:**
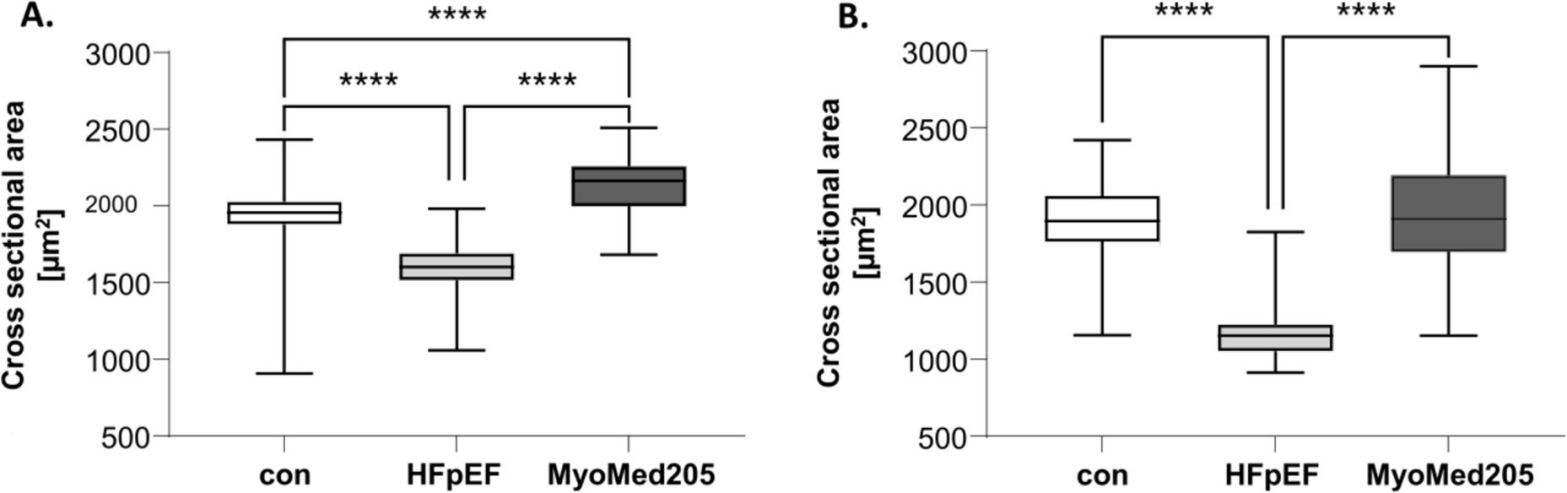
MyoMed205 has antiatrophic effects. CSA of (A) soleus and (B) EDL from ZSF1‐control (con) and untreated ZSF1‐HFpEF rats (HFpEF) as well as treated ZSF1‐HFpEF rats (MyoMed205) at 32 weeks of age. The results are expressed as median ± Min–Max (*n* = 4–5 per group). **** *p* < 0.0001.

With respect to treatment with MyoMed205, we observed significant regression of myofiber atrophy in the Sol and EDL. Analysing SKM force, earlier experiments revealed an increase of specific force after MyoMed205 treatment, compared with untreated HFpEF rats. In particular, the EDL significantly increased by 8%, whereas the Sol showed minor improvement compared with untreated rats [5].

### 3.4 Expression and Secondary Modifications of Titin in HFpEF and Attenuation by MyoMed205

**FIGURE 3:**
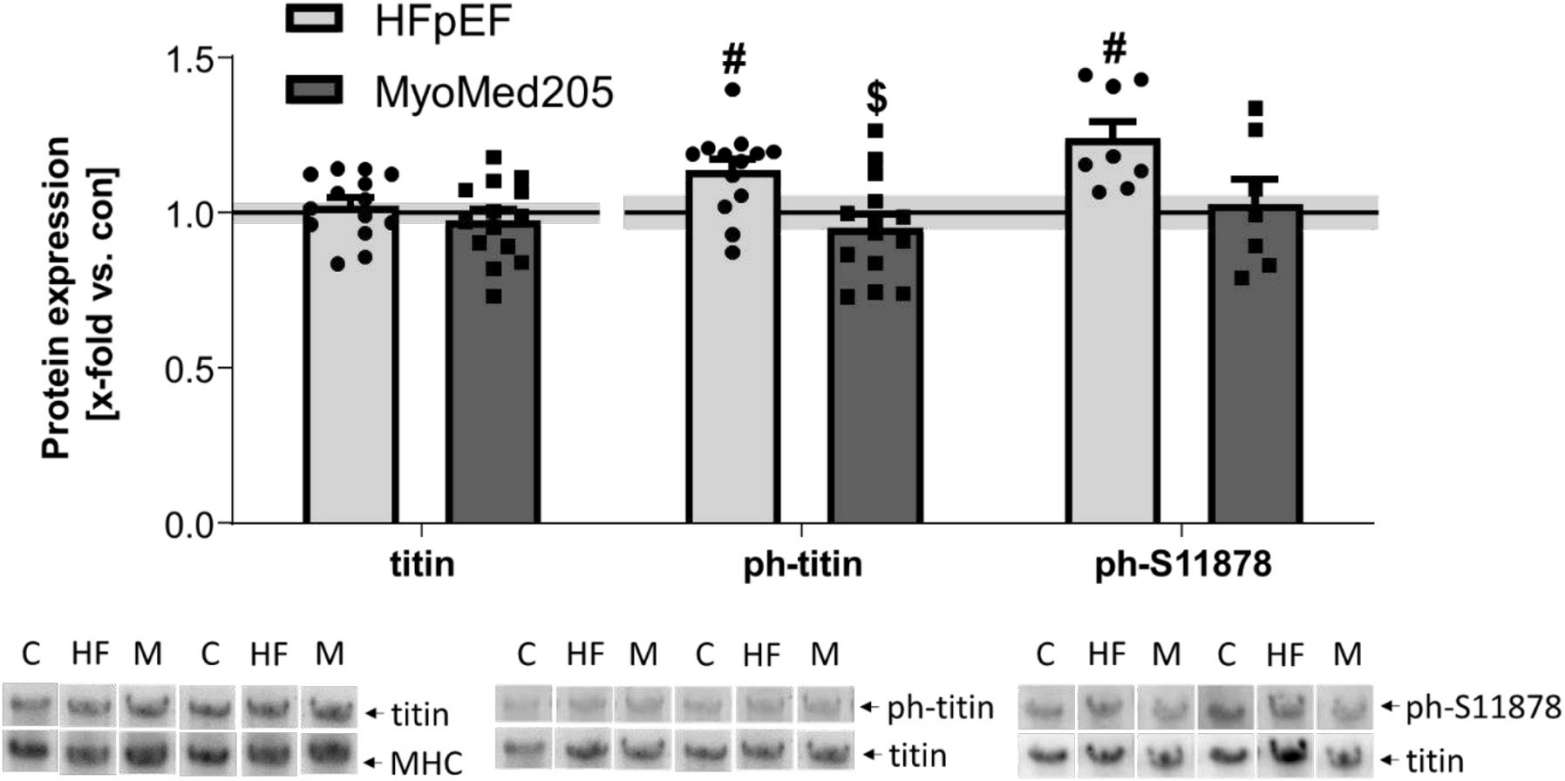
Titin hyperphosphorylation in the Sol can be normalized by treatment with MyoMed205. Titin expression, as well as phosphorylation, was quantified via VAGE analysis in skeletal muscle homogenates of Sol obtained from ZSF1‐control (con) (represented as a solid line) and untreated ZSF1‐HFpEF rats (HFpEF) as well as treated ZSF1‐HFpEF rats (MyoMed205) at 32 weeks of age. The results are expressed as x‐fold versus control (solid line, set to 1.0) ± SEM (*n* = 8–14 per group). Representative stains and VAGE blots are depicted below (c = con, HF = HFpEF, M = MyoMed205). # *p* < 0.05 versus con, $ *p* < 0.05 versus HFpEF.

**FIGURE 4:**
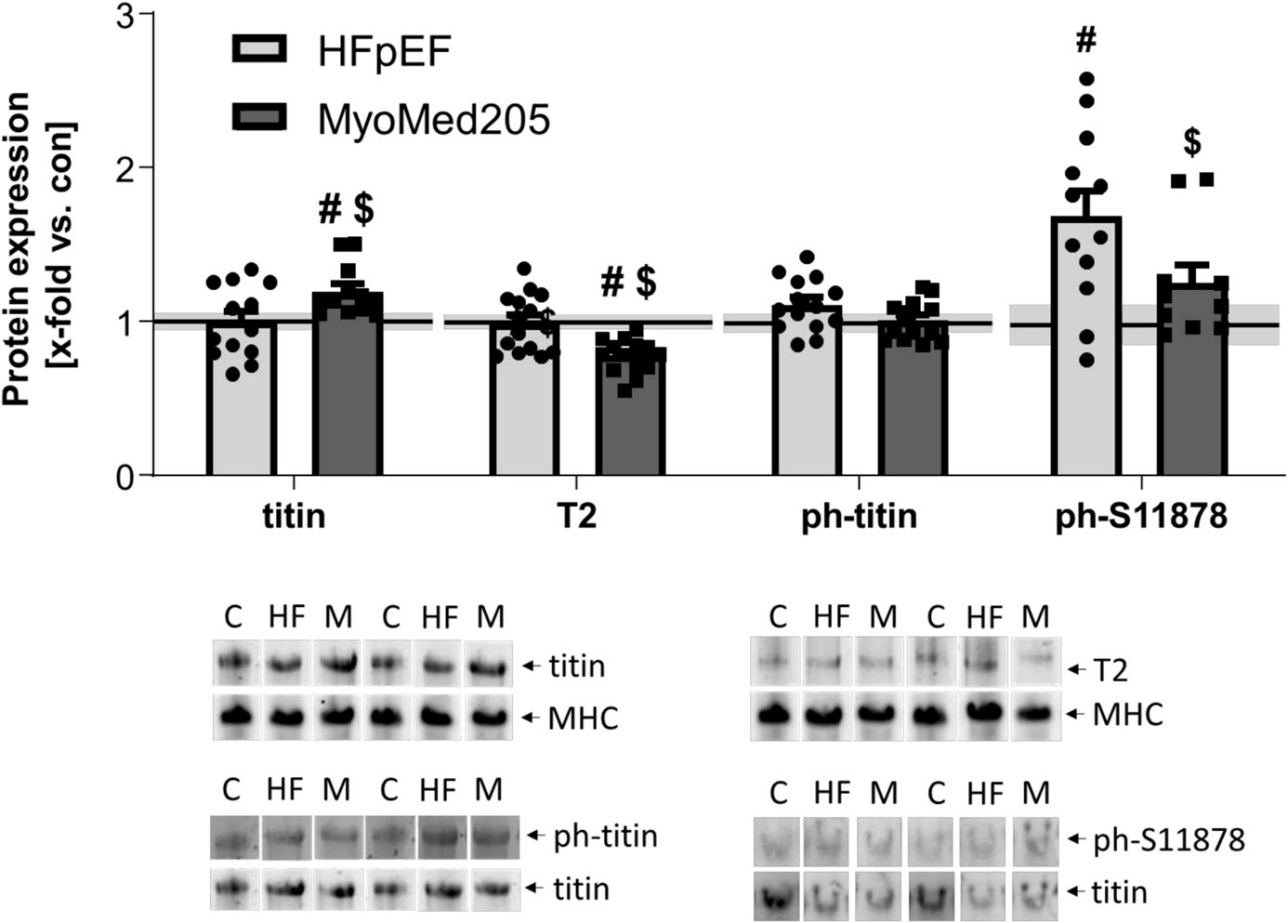
Titin degradation gets reduced, whereas hyperphosphorylation can be normalized by treatment with MyoMed205 in the EDL. Protein expression, as well as phosphorylation of titin, was quantified via VAGE analysis in SKM homogenates of EDL obtained from ZSF1‐control (con) (represented as a solid line) and untreated ZSF1‐HFpEF rats (HFpEF) as well as treated ZSF1‐HFpEF rats (MyoMed205) at 32 weeks of age. The results are expressed as x‐fold versus control (solid line, set to 1.0) ± SEM (*n* = 10–14 per group). Representative stains and VAGE blots are depicted below (c = con, HF = HFpEF, M = MyoMed205). # *p* < 0.05 versus con, $ *p* < 0.05 versus HFpEF.

We checked titin modifications and changes in protein expression in the Sol and the EDL of our animals. In terms of total titin expression, no change was evident in any of the groups. Interestingly, we were not able to detect T2, the main splice isoform of titin, in any of our Sol samples (Figure 3). In the EDL, we could distinguish between the full‐length titin and the spliced T2 isoform. Although neither titin nor T2 expression changed during HFpEF development, treatment with MyoMed205 led to increased full‐length titin expression, accompanied by a decrease in T2 levels. These changes of protein abundance are negatively correlated with each other (*r* = −0.47, *p* = 0.004) (Figure 4). In terms of secondary modifications, a significant titin hyperphosphorylation was evident in the Sol of ZSF1‐HFpEF rats, which was significantly reversed by treatment with MyoMed205 (Figure 3). The EDL, on the other hand, only showed numerically slightly increased phosphorylated titin levels (*p* = 0.09) in the diseased animals (Figure 4). By analysing specific phosphorylation sites, we focused on S11878 phosphorylation, which is located at the PEVK region of titin and is strongly associated with titin stiffness [31]. For this specific site, we detected in both muscles a significantly increased phosphorylation level in HFpEF rats, which was reduced to a non‐significant level, compared with control rats, by MyoMed205.

**FIGURE 5:**
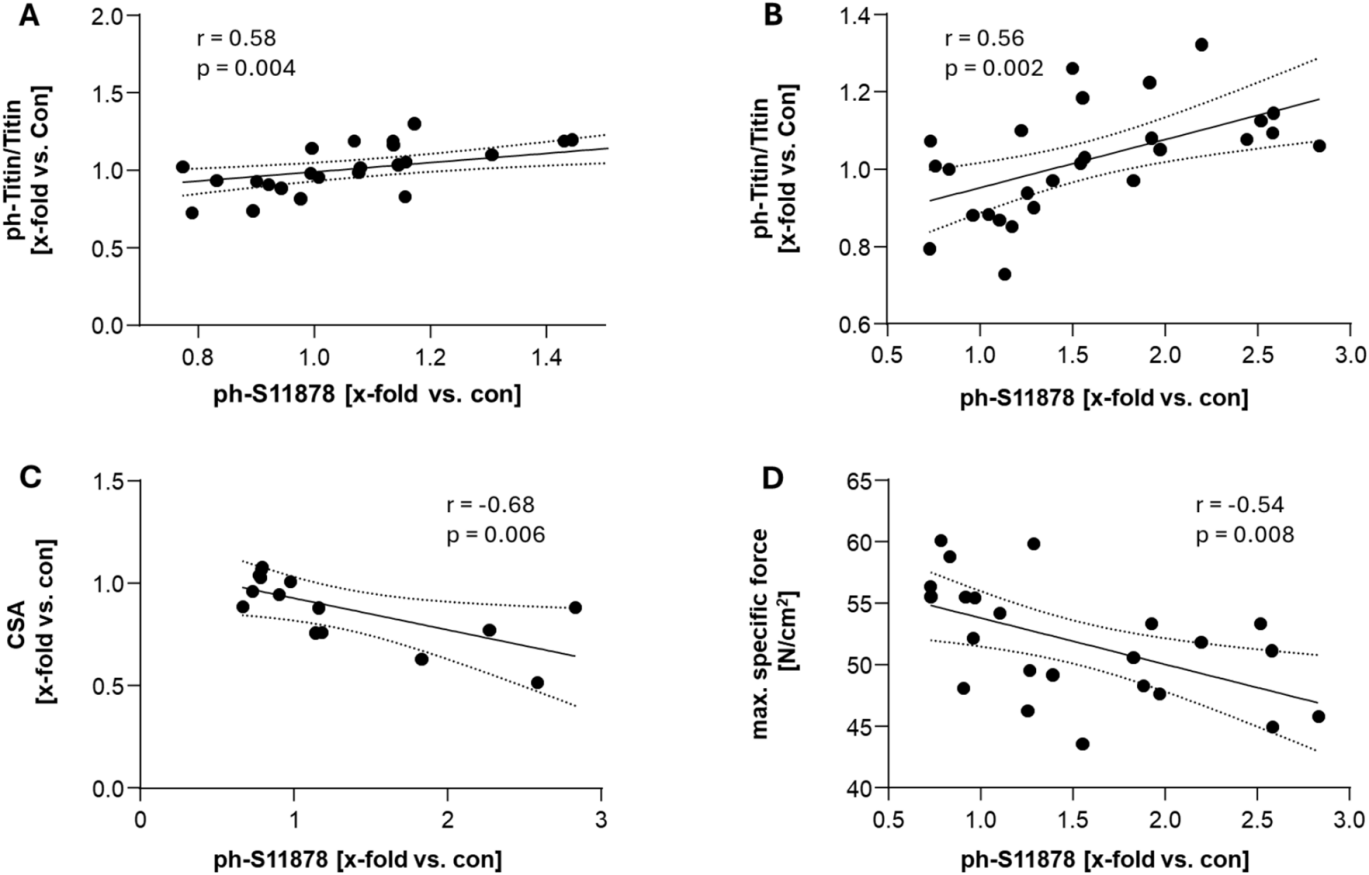
PEVK phosphorylation strongly correlates with functional parameters in the SKM. Correlation analyses between S11878 phosphorylation and total titin phosphorylation in the Sol (A) and the EDL (B). In addition, a correlation between S11878 phosphorylation and SKM CSA (C) and maximal (max.) specific force in the EDL (D) was evident.

For both muscles, a positive correlation between site‐specific S11878 and total phosphorylation was evident (Sol: *r* = 0.58, *p* = 0.004, *n* = 33; EDL: *r* = 0.56, *p* = 0.002, *n* = 33) (Figure 5A,B), and an inverse correlation between S11878 titin phosphorylation and SKM CSA was observed (*r* = −0.68, *p* = 0.006, *n* = 20) (Figure 5C). Additionally, in the EDL, but not in the Sol, an inverse correlation between S11878 phosphorylation and the maximum specific force (*r* = −0.54, *p* = 0.008, *n* = 28) was evident (Figure 5D).

With respect to nebulin, no changes were detected between the groups (data not shown). Based on our findings showing greater pathological changes in EDL as in the Sol, we directed most of the subsequent analyses towards the EDL.

### 3.5 MHC Expression and Protein Ubiquitination in HFpEF and Attenuation by MyoMed205 in the EDL (Figure 6A)

**FIGURE 6:**
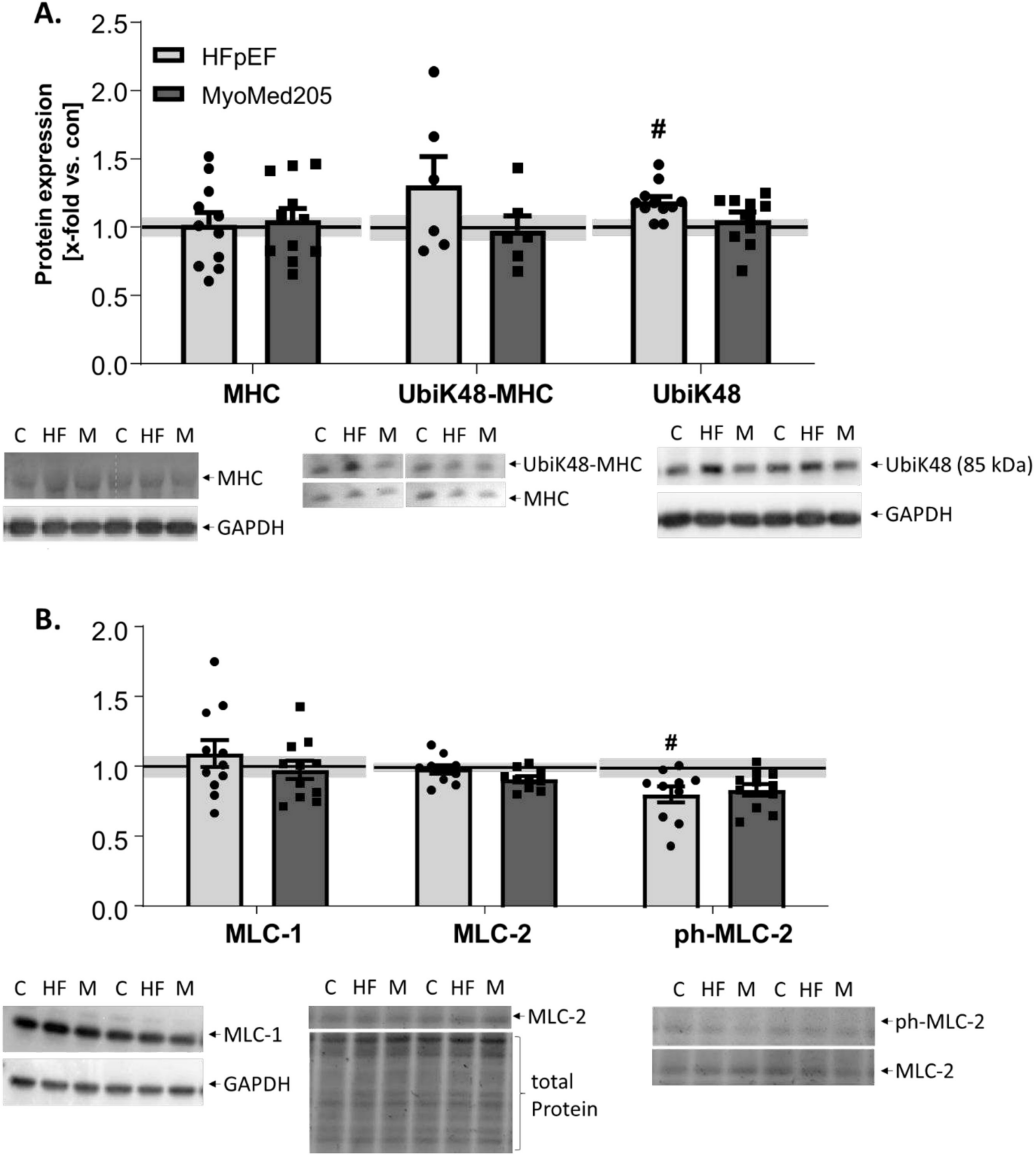
Ubiquitination, but not of MHC, is susceptible to MyoMed205, whereas MLC phosphorylation is significantly decreased only in HFpEF animal. MHC expression as well as ubiquitination of MHC and a 85‐kDa protein (A) and MLC isoform expression and phosphorylation (B) were quantified by WB analysis in skeletal muscle homogenates of EDL, obtained from ZSF1‐control (con) (represented as solid line) and untreated ZSF1‐HFpEF rats (HFpEF) as well as treated ZSF1‐HFpEF rats (MyoMed205) at 32 weeks of age. The results are expressed as x‐fold versus control (solid line, set to 1.0) ± SEM (*n* = 6–11 per group). Representative stains and blots are depicted below (c = con, HF = HFpEF, M = MyoMed205). # *p* < 0.05 versus con, $ *p* < 0.05 versus HFpEF.

Total MHC expression, as well as MHC ubiquitination, did not differ significantly between any of the groups. However, when analysing the ubiquitination of the most prominent protein band (~85 kDa) from EDL, a significant increase was observed in the HFpEF rats. This increase could be reversed by MyoMed205 treatment. Additionally, the phosphorylation levels of MHC were not changed by either the disease or the treatment (data not shown).

### 3.6 MLC Modulation in HFpEF and Attenuation by MyoMed205 in the EDL (Figure 6B)

Neither MLC‐1 nor MLC‐2 expression was significantly altered in any of the groups. Nevertheless, with respect to phosphorylation levels of MLC‐2, we saw a significant decrease in the diseased animals. Treatment with MyoMed204 slightly increased the phosphorylation to a non‐significant level.

### 3.7 Expression of Proteins Involved in Sarcomere Organization in HFpEF and Attenuation by MyoMed205 in the EDL (Figure S1)

Titin is part of the sarcomere, a large complex of proteins. Some of these proteins are important for structural organization. Four of them were analysed in our study. The expression of α‐actinin and myotilin was not significantly altered between ZSF‐1 control and untreated HFpEF rats. Unexpectedly, MyoMed205‐treated HFpEF rats showed a numerically decreased expression to the diseased group regarding α‐actinin (*p* = 0.05) and to the control group concerning myotilin (*p* = 0.06). Neither SMYD2 nor telethonion expression was altered in either of the animals. When analysing protein expression of α‐sarcomeric actin in the EDL, no significant difference was observed between the groups (data not shown).

### 3.8 Expression of Ca^**2**+^‐Related Proteins in HFpEF and Attenuation by MyoMed205 in the EDL (Figure 7)

**FIGURE 7:**
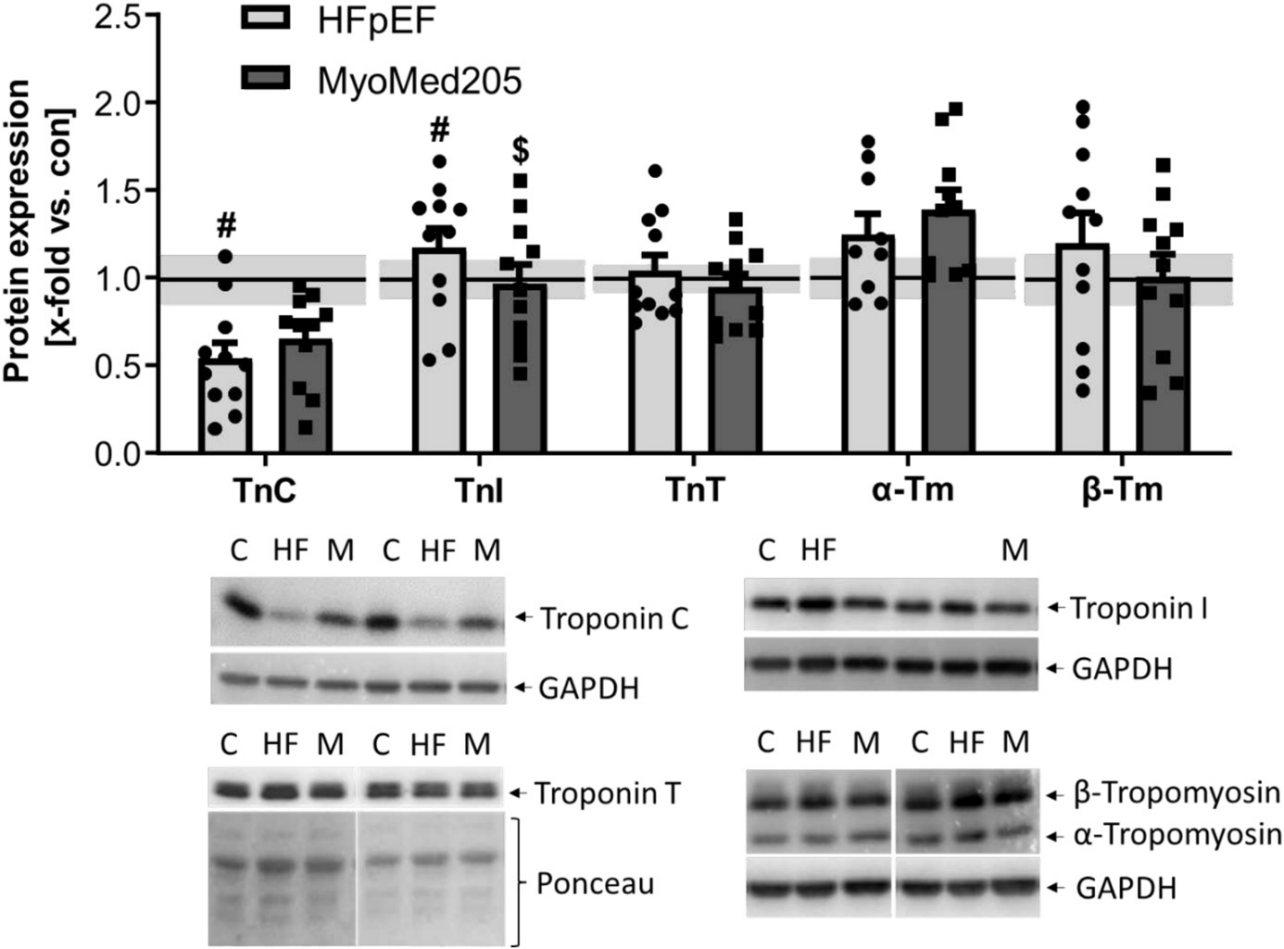
MyoMed205 modulates expression of proteins associated with Ca^**2**+^‐dependent muscle contraction. TnC, TnI, TnT and α‐ and β‐Tm expression was quantified by WB analysis of skeletal muscle homogenates of EDL obtained from ZSF1‐control (con) (represented as solid line) and untreated ZSF1‐HFpEF rats (HFpEF) as well as treated ZSF1‐HFpEF rats (MyoMed205) at 32 weeks of age. The results are expressed as x‐fold versus control (solid line, set to 1.0) ± SEM (*n* = 10–14 per group). Representative blots are shown below (c = con, HF = HFpEF, M = MyoMed205). # *p* < 0.05 versus con, $ *p* < 0.05 versus HFpEF.

Calcium handling is critical for supporting muscle contraction. The troponin–tropomyosin complex, which is a part of the sarcomere, regulates this process. Troponin C (TnC) expression was significantly decreased in HFpEF and could be slightly elevated by treatment with MyoMed205. In contrast, troponin I (TnI) expression was significantly upregulated in HFpEF and could be reduced to control levels by MyoMed205 treatment. Troponin T (TnT) expression was not altered in any of the groups. Expression of tropomyosin isoforms (Tm) was not altered in the HFpEF rats, but α‐Tropomyosin was slightly increased in the treated group (*p* = 0.07).

### 3.9 Expression of Muscle Atrophy Markers in HFpEF and Attenuation by MyoMed205 (Figure 8)

**FIGURE 8:**
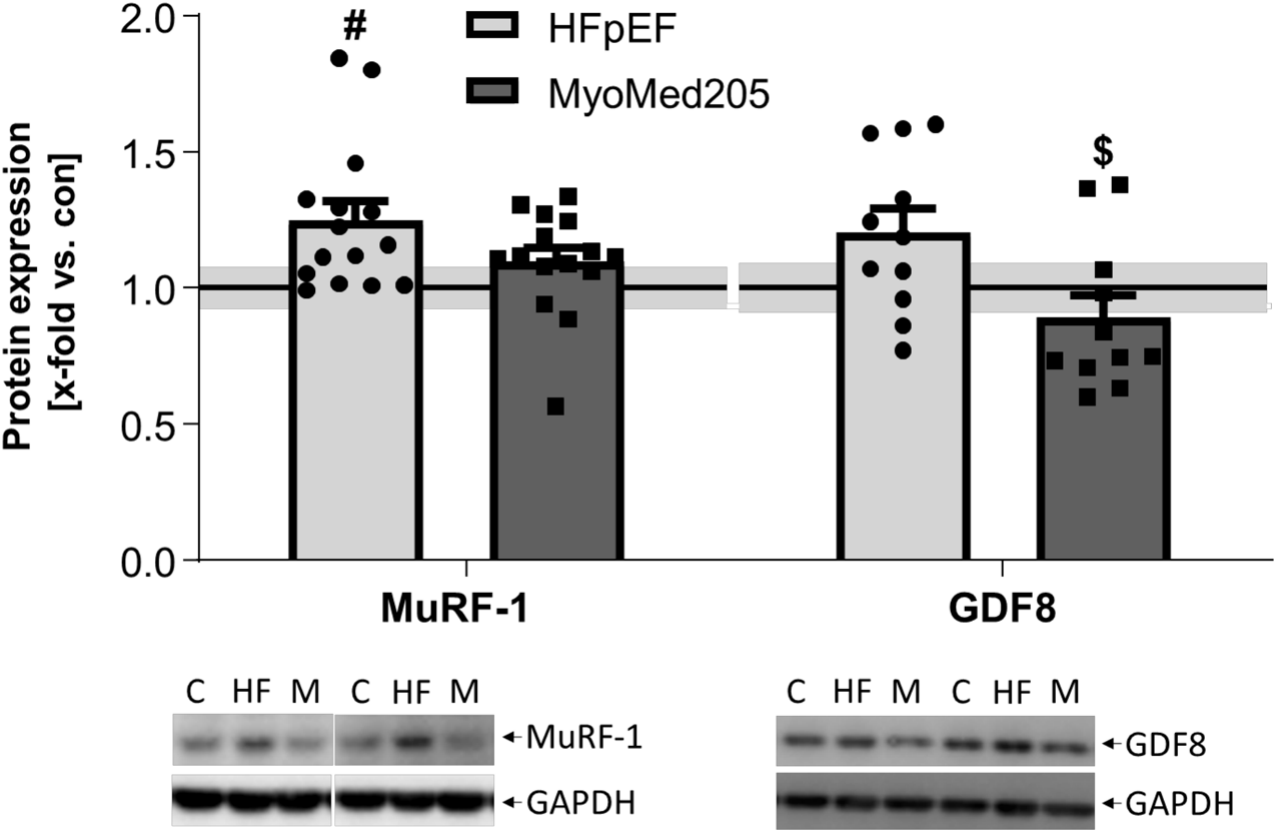
Normalized expression of atrophy markers after treatment with MyoMed205. MuRF1 and GDF8 expression was quantified via WB analysis of skeletal muscle homogenates of EDL obtained from ZSF1‐control (con) (represented as solid line) and untreated ZSF1‐HFpEF rats (HFpEF) as well as treated ZSF1‐HFpEF rats (MyoMed205) at 32 weeks of age. The results are expressed as x‐fold versus control (solid line, set to 1.0) ± SEM (*n* = 10–14 per group). Representative blots are shown below (c = con, HF = HFpEF, M = MyoMed205). # *p* < 0.05 versus con, $ *p* < 0.05 versus HFpEF.

Proteins such as MuRF1, muscle atrophy F‐box (MAFbx), four and a half LIM domain 1 (FHL1) and myostatin (GDF8) serve as markers for accelerated muscle atrophy. Although FHL1 and MAFbx expression did not differ among the three groups (data not shown), MuRF1 was significantly upregulated under HFpEF, and treatment with MyoMed205 numerically decreased MuRF1 levels (*p* = 0.09) compared with untreated HFpEF rats. GDF8 was slightly upregulated (*p* = 0.09) in diseased rats compared with controls but significantly reduced by MyoMed205 treatment.

## 4 Discussion

Exercise intolerance, which is partly due to SKM alterations, is one of the major hallmarks of HFpEF, affecting quality of life and prognosis [1, 2]. Besides alterations like muscle atrophy, fibre‐type switching and reduced energy supply, we recently reported titin hyperphosphorylation in the SKM [14]. Titin is known to be a major regulator of muscle development and function [32]. MuRF1, an important protein for controlling SKM atrophy, is an interaction partner of titin and is thought to mark it through K48 ubiquitination for degradation [20]. In the present study, we investigated the effects of MyoMed205, a small molecule that has shown potential to inhibit the interaction between MuRF1 and titin, on titin and contractile proteins in an established animal model of HFpEF. The effects of MyoMed205 in HFpEF can be summarized as follows:

- Titin hyperphosphorylation, driven in part by S118787 phosphorylation, is evident in slow as well as fast SKM and can be reversed by MyoMed205 treatment.
- Titin S11878 phosphorylation correlates with muscle force and atrophy in the SKM.
- MyoMed205 normalized protein ubiquitination, attenuated MLC phosphorylation and reduced titin degradation
- Dysregulation of proteins related to atrophy and Ca^2+^‐dependent muscle contraction can be reversed by MyoMed205 treatment in the EDL.

Taken together, our findings revealed improved SKM function and a remodelling of proteins relevant for controlling muscle contraction by MyoMed205. Besides the modulation of mitochondrial function [5], these reported alterations in titin, secondary modifications and sarcomere proteins may explain the beneficial effect of MyoMed205 in the setting of HFpEF.

### 4.1 Titin Hyperphosphorylation Can Be Reversed by MyoMed205 Treatment

We detected titin hyperphosphorylation in fast and slow SKM. These results demonstrate that the increased phosphorylation levels are not muscle type specific but can be detected ubiquitously in the SKM of HFpEF animals, although we detected time‐dependent differences between EDL and Sol. Nevertheless, when looking at the SKM type–specific phosphorylation patterns, we may conclude that the EDL is more prone to SKM alterations/dysfunction in HFpEF when compared with the Sol.

With respect to site‐specific phosphorylation, we detected that titin hyperphosphorylation is mainly located at S11878, a serine residue in the PEVK region of titin [31]. Since phosphorylation at the PEVK region is known to stiffen the sarcomere and to impair muscle function [31], our results suit the specific force development in the different muscles during disease, as well as their response to treatment. When correlating muscle force with titin S11878 phosphorylation, we again found significant differences between fast and slow SKM. These results may suggest that in the Sol, titin and its modulation have less of an impact on regulating SKM force development when compared with the EDL. This may be due to the Sol being a slow‐twitch muscle with higher oxidative capacity, depending on its mitochondrial energy supply, which is impaired in HFpEF, leading to decreased performance. The EDL on the other hand is a fast‐twitch muscle and has increased reliance on anaerobic glycolysis, which may be more dependent for sarcomeric function and titin compliance [33, 34].

Another point of interest is the relationship between atrophy and hyperphosphorylation in the EDL and Sol, which has already been discussed in the literature [35, 36, 37]. Taken together, our data show that PEVK phosphorylation has strong negative effects on muscle function, more prominent in the EDL, which could be partly reversed by MyoMed205 treatment.

As already described in the current literature [38], in the Sol, we were unable to distinguish between full‐length titin and the spliced T2 isoform, which is a degradation product of titin [39]. This finding argues for different degradation processes between EDL and Sol, since the EDL exhibited both titin forms, which were inversely correlated with each other.

### 4.2 Protein, but Not MHC Ubiquitination, Is Positively Influenced by MyoMed205

Regarding total protein ubiquitination, earlier experiments determined increased K48 ubiquitination in the TA of HFpEF rats, which was normalized after MyoMed205 treatment [5]. Considering that MyoMed205 is supposed to inhibit MuRF1‐dependent ubiquitination, this normalization was expected and could be confirmed for an unknown protein band in the EDL in the present study. For MHC, no influence of MyoMed205 could be detected. Since MHC expression is supposed to be regulated by MuRF1 levels [40], we suggest that MyoMed205 is not influencing this interaction.

With respect to titin ubiquitination, we did not investigate ubiquitin levels, since earlier studies have shown that cardiac titin ubiquitination is not altered in MuRF1 KO mice [23]. Nevertheless, the expression pattern of titin in MyoMed205‐treated animals revealed that the titin degradation process is disturbed, since elevated levels of full‐length titin and decreased levels of its degradation product T2 were detected.

### 4.3 MLC Cross‐Bridge Binding Is Impaired in HFpEF in the EDL and Can Be Slightly Normalized by MyoMed205 Treatment

MLC‐2 (also myosin regulatory light chain [MRLC] [30]) phosphorylation is known to regulate muscle function, by enhancing the actin–myosin binding, as well as the calcium‐sensitivity and to be protective against heart dysfunction [30, 41, 42]. The results of our study clearly underline these findings and furthermore show that treatment with MyoMed205 may have a positive impact on muscle function shown by the slight increase of the phosphorylation, even though the contraction is primarily regulated by the troponin–tropomyosin interaction [41].

### 4.4 Altered Expression of Muscle Contraction Proteins and Atrophy Markers Can Be Reversed by MyoMed205

MuRF1 upregulation is associated with atrophy, which has been shown in different disease patterns [43, 44, 45]. With respect to protein expression over time, a study investigating the effects of hindlimb immobilization over 6 days on the Sol revealed a consistent increase in MuRF1 expression over the first few days, which decreased to a less significant level by the end of the observation period [45]. This expression pattern was also observed in our HFpEF rats, with the highest expression of MuRF1 in 20‐week‐old animals (Figure S1B). The increased expression of MuRF1 in 32‐week‐old MyoMed205‐treated rats may be a physiological response, since the biological system recognizes that MuRF1‐dependent ubiquitination is disturbed and starts counteracting by the upregulation of other E3 ligases, such as MuRF‐3 and CHIP, or proteases [46].

Additionally, GDF8, also known as myostatin, is a member of the growth factor‐β superfamily and is known as a negative regulator of SKM growth [47]. Our observed expression changes of GDF8 may be one reason for the reduction of CSA in the EDL.

Regarding functional muscle properties, the Tn–Tm complex is a very important element in terms of Ca^2+^‐dependent contraction. Every part of this complex has a different task, from sensing Ca^2+^ to initiating and performing the muscle contraction. The three troponins, as well as Tm, are always expressed in equal ratios [48]. All three troponins express different isoforms, depending on the fibre type [49]. TnC initiates the contraction and is barely a limiting factor but rather a Ca^2+^‐sensing switch. TnI inhibits actomyosin ATPase and leads to muscle relaxation. TnT connects the troponin complex to the Tm‐dimers, which are located around the thin filament [49]. It is well described that preferentially αα‐ or αβ‐dimers are formed, since ββ‐dimers are known to be less stable [50]. Additionally, TnT is responsible for transducing conformational changes of TnC to Tm, thereby regulating muscle relaxation and contraction [49]. These changes, induced by the troponin complex, lead to different localization of Tm, preventing or permitting contraction of the sarcomere [S1]. In our study, we detected the expression of the slow TnC‐isoform and TnI's fast‐isoform. Our findings may indicate a greater extent of the Tn–Tm complex, whereas the expression changes of fibre type–specific isoforms advert a shift to fast fibres in untreated HFpEF rats. This fibre type shift has already been reported to occur in HFpEF, limiting exercise performance of the muscles [S2], since slow fibres are more fatigue‐resistant and show a greater force production [49]. These findings argue for improved exercise capacity after MyoMed205 treatment because of increased Tn–Tm complex expression, as well as a reversal of the fibre type shift towards slow fibres.

### 4.5 Sarcomere Organization Is Not Impaired in the EDL in HFpEF

The sarcomere, which is responsible for muscle contraction, is divided into A‐band, I‐band and M‐line. Although the I‐band contains thin filament (actin), the thick filament (myosin) is located in the A‐band, which is bisected by the M‐line. The thick filament aligns with the rest of the sarcomere by interacting with titin [S3]. These sarcomere structures are aligned between two z‐discs [S4]. The z‐disc is composed of thin filaments that are cross‐linked by α‐actinin and connect to different proteins, such as nebulin and myotilin, as well as titin, which is bound through telethonin and α‐actinin [S5].

Since α‐actinin and myotilin are closely related to each other [S6, S7], the simultaneous decrease in expression that we detected seems logical, but the reason remains unclear. As both proteins are tremendously important for z‐disc structure and function [S7], this could be interpreted as a sign of muscular dystrophy. On the other hand, these findings could suggest sarcomere remodelling because of protein mutations occurring in HFpEF, which need to be removed. Additionally, it is possible that alternative proteins, such as telethonin and myopalladin, assume some functions of myotilin and that another isoform of α‐actinin, α‐actinin‐3, which is associated with high performance of fast fibres, takes on the z‐disc assembly.

## 5 Study Limitations

Our work provides an initial foundation that may allow future work to develop therapeutic targets that could potentially be used to counteract symptoms of HFpEF and to improve patients' quality of life. However, some limitations must be addressed.

First, our study was performed in ZSF1‐HFpEF rats. Although this is a validated metabolic animal model for HFpEF, it does not reproduce the complete human condition, including the degree of muscle myopathy. Therefore, the model may not be transferable to other forms of HFpEF such as purely hypertensive‐triggered HFpEF, as in Dahl‐salt–sensitive rats. This problem needs to be addressed in further studies. However, an earlier study identified the ZSF1 model as the most suitable one for examining alterations of the SKM in HFpEF when compared with patient responses [S8].

Additionally, only female rats were used in the present study. Therefore, a generalization to the total HFpEF population is not possible. Nevertheless, registry and community‐based studies have shown that the majority of HFpEF patients are elderly women exhibiting a variety of comorbidities, such as hypertension, diabetes, pulmonary disease, chronic kidney disease and obesity [S9, S10].

Second, studies have shown that a complete inactivation of MuRF genes is cardiotoxic in mice over time [S11]. The present study used a small molecule, supposed to interfere with target recognition of MuRF1 and treated rats over a time of 12 weeks. Regarding treatment of patients over periods exceeding 1‐year, possible side effects may exclude the application of MyoMed205 in HFpEF patients. The examination of toxicology data from more anthropoid animal models may be the groundwork for human trials and is urgently needed.

## 6 Conclusion

In conclusion, the results of the present study reveal that MyoMed205 reduces hyperphosphorylation of titin, which was associated with attenuated contractile dysfunction and atrophy, in an established animal model of HFpEF. MyoMed205 primarily affects the S11878 phospho‐site in the PEVK region of titin, which is associated with titin stiffening. A correlation between S11878 titin phosphorylation and muscle force, as well as atrophy, supports the hypothesis that MyoMed205 modulates titin phosphorylation and thereby improves muscle function and reduces atrophy. Nevertheless, future experiments using cell culture and specific knockout animals need to be performed to validate this relationship. The study also strengthens the notion that fast as well as slow muscle fibres benefit from MyoMed205 regarding titin phosphorylation although some time and muscle‐specific differences were detected.

## Ethics Statement

The study was conducted according to the guidelines of the Declaration of Helsinki and the guidelines of the local animal ethics committee. All experiments and procedures were approved by the local animal research council, TU Dresden and the Landesbehörde Sachsen (TVV 42/2018).

## Consent

The authors have nothing to report.

## Conflicts of Interest

V. A. and S. L. report a patent for MyoMed‐205, ID#704946 and further derivatives for its application to chronic muscle stress states (patent accession No. WO2021023643A1). B. V., A. S., A. A., M. E. P. J., P. B.; A. M. and T. S. B. declare no conflicts of interest. N. M. reports personal fees from Edwards Lifesciences, Medtronic, Biotronik, Novartis, Sanofi Genzyme, AstraZeneca, Pfizer, Bayer, Abbott, Abiomed and Boston Scientific, outside the submitted work. A. L. reports grants from Novartis, personal fees from Medtronic, Abbott, Edwards Lifesciences, Boston Scientific, Astra Zeneca, Novartis, Pfizer, Abiomed, Bayer, Boehringer and other from Picardia, Transverse Medical, Claret Medical, outside the submitted work. The funders had no role in the design of the study; in the collection, analyses or interpretation of data; in the writing of the manuscript; or in the decision to publish the results.

## Supporting information


**Figure S1** Sarcomere organization seems to be slightly perturbed by MyoMed205 treatment.

**Figure S2** MurRF‐1 expression is significantly increased in 20‐ and 32‐week‐old animals.

**Data S1** Supporting information.

## Data Availability

The data that support the findings of this study are available from the corresponding author upon reasonable request.
