# Supplementary material for: MyoMed205 Counteracts Titin Hyperphosphorylation and the Expression of Contraction‐Regulating Proteins in a Rat Model of HFpEF
Source: J Cachexia Sarcopenia Muscle. 2025 Jun 4;16(3):e13843. doi: 10.1002/jcsm.13843 (PMC12134774; doi:10.1002/jcsm.13843)
Supplement: Supplementary file 1 — Figure S1 Sarcomere organization seems to be slightly perturbed by MyoMed205 treatment. [file JCSM-16-e13843-s001.pdf]

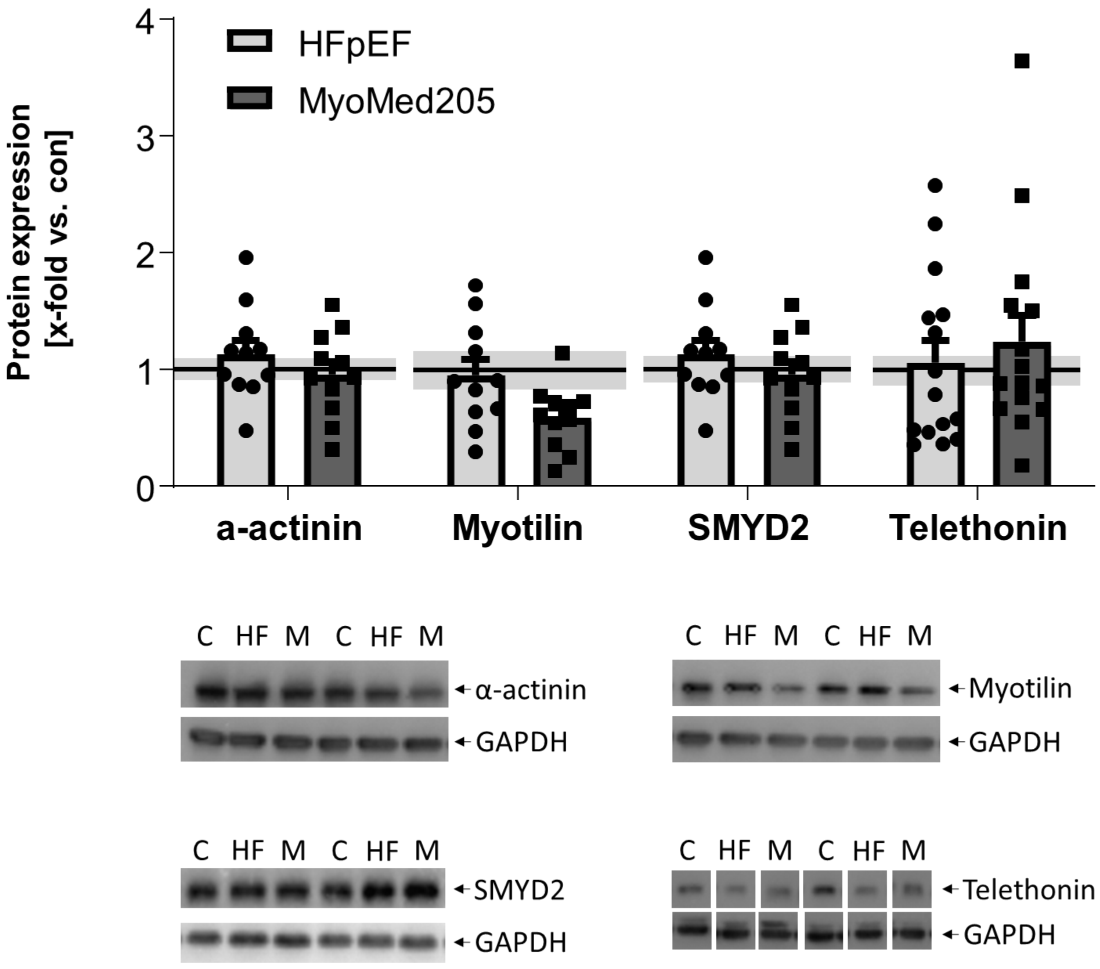

Supplemental Figure 1 - Sarcomere organization seems to be slightly perturbed by MyoMed205 treatment. α-actinin, myotilin, SMYD2 and telethonin expression was quantified via WB analysis in skeletal muscle homogenates of EDL, obtained from ZSF1-control (con) (represented as scattered line) and untreated ZSF1-HFpEF rats (HFpEF) as well as treated ZSF1-HFpEF rats (MyoMed205) at 32 weeks of age. The results are expressed as x-fold vs. control (scattered line, set to 1.0) ± SEM (n = 10–15 per group). Representative blots are shown below (c = con, HF = HFpEF, M = MyoMed205).
