# Supplementary material for: MyoMed205 Counteracts Titin Hyperphosphorylation and the Expression of Contraction‐Regulating Proteins in a Rat Model of HFpEF
Source: J Cachexia Sarcopenia Muscle. 2025 Jun 4;16(3):e13843. doi: 10.1002/jcsm.13843 (PMC12134774; doi:10.1002/jcsm.13843)
Supplement: Supplementary file 2 — Figure S2 MurRF‐1 expression is significantly increased in 20‐ and 32‐week‐old animals. [file JCSM-16-e13843-s003.pdf]

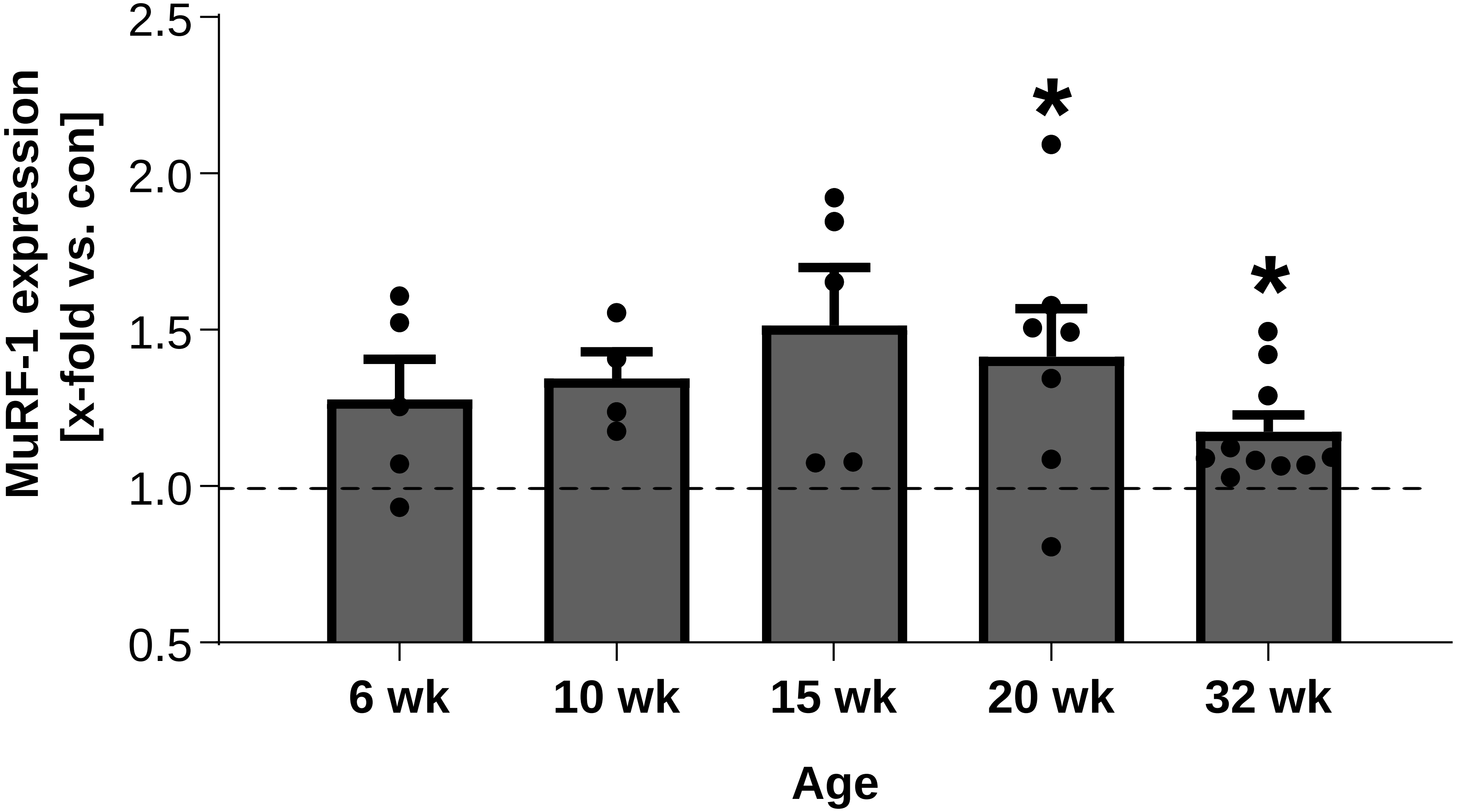

Supplemental Figure 2 – MuRF-1 expression is significantly increased in 20- and 32-week-old animals. MuRF-1 expression was quantified via WB analysis of skeletal muscle homogenates of Sol obtained from 6-, 10-, 15-, 20- and 32-week-old (wk) ZSF-1-control (con) (represented as scattered line) and ZSF1-HFpEF rats (HFpEF). The results are expressed as x-fold change vs. control control (scattered line, set to 1.0) ± SEM (n = 5–10 per group).

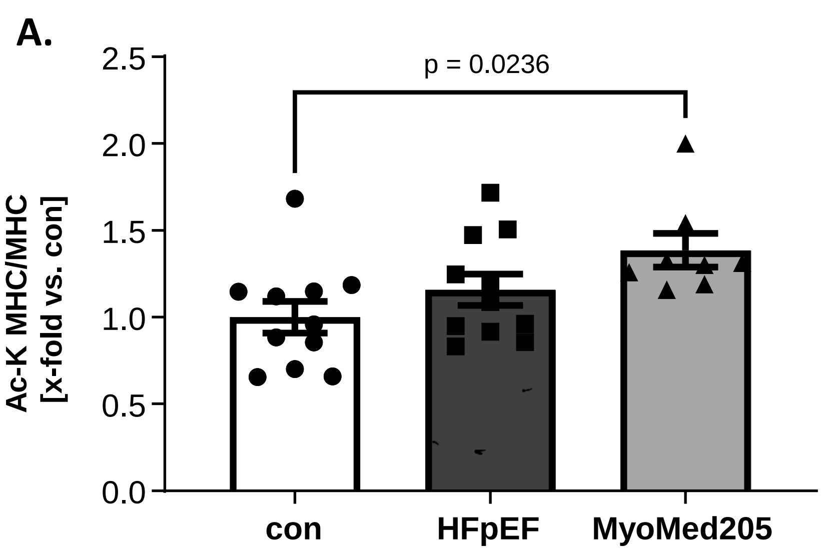
A.
p = 0.0236
Ac-K MHC/MHC
[x-fold vs. con]
2.5
2.0
1.5
1.0
0.5
0.0
con
HFpEF
MyoMed205

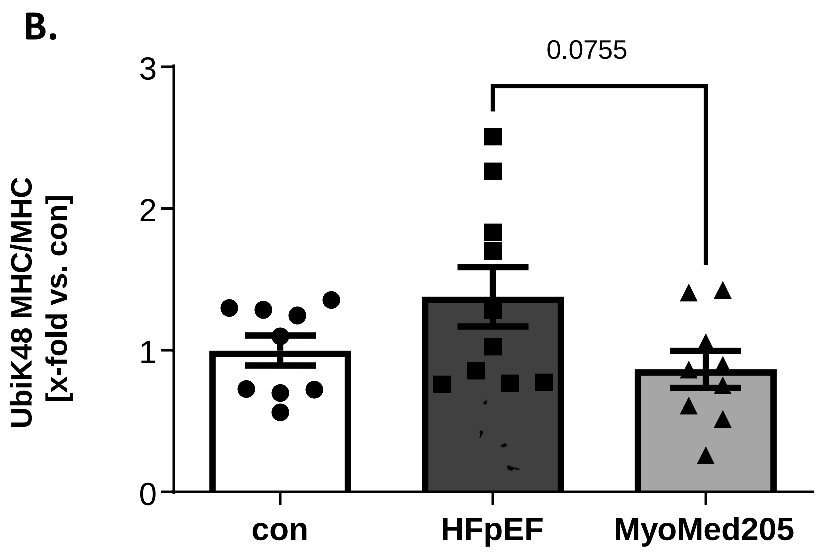
B.
0.0755
UbiK48 MHC/MHC
[x-fold vs. con]
3
2
1
0
con
HFpEF
MyoMed205

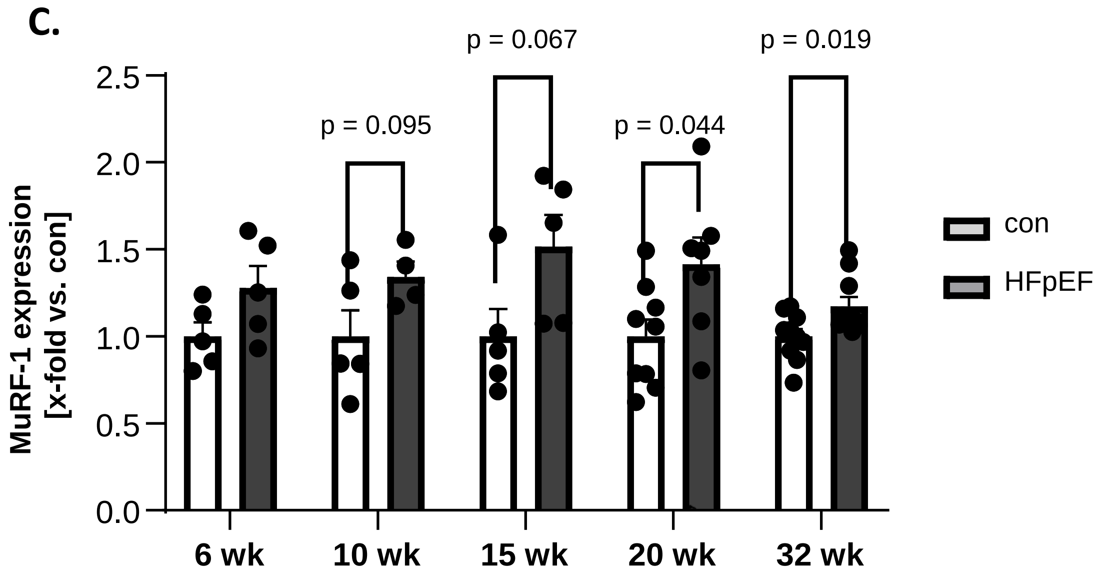
C.
p = 0.095
p = 0.067
p = 0.044
p = 0.019
MuRF-1 expression
[x-fold vs. con]
2.5
2.0
1.5
1.0
0.5
0.0
6 wk
10 wk
15 wk
20 wk
32 wk
con
HFpEF
