# Supplementary material for: MyoMed205 Counteracts Titin Hyperphosphorylation and the Expression of Contraction‐Regulating Proteins in a Rat Model of HFpEF
Source: J Cachexia Sarcopenia Muscle. 2025 Jun 4;16(3):e13843. doi: 10.1002/jcsm.13843 (PMC12134774; doi:10.1002/jcsm.13843)
Supplement: Supplementary file 3 — Data S1 Supporting information. [file JCSM-16-e13843-s002.pdf]

Supplemental References

S1. A. V. Gomes, J. D. Potter, and D. Szczesna-Cordary, IUBMB life **54**, 6 (2002).

S2. E. L. Saw, S. Ramachandran, M. Valero-Muñoz, and F. Sam, Current opinion in cardiology **36**, 2 (2021).

S3. *Cold Spring Harbor monograph series*, Vol. 33: *C. Elegans*, Ed. by D. L. Riddle, T. Blumenthal, B. J. Meyer, and J. R. Priess (Cold Spring Harbor Laboratory Press, Cold Spring Harbor, NY, 1997) [eng].

S4. C. A. Henderson, C. G. Gomez, S. M. Novak, L. Mi-Mi, and C. C. Gregorio, Comprehensive Physiology **7**, 3 (2017).

S5. Z. Wang, M. Grange, T. Wagner, A. L. Kho, M. Gautel, and S. Raunser, Cell **184**, 8 (2021).

S6. P. Salmikangas, O. M. Mykkänen, M. Grönholm, L. Heiska, J. Kere, and O. Carpén, Human molecular genetics **8**, 7 (1999).

S7. K. Wadmore, A. J. Azad, and K. Gehmlich, International journal of molecular sciences **22**, 6 (2021).

S8. K. Goto, A. Schauer, A. Augstein, M. Methawasin, H. Granzier, M. Halle, E. M. van Craenenbroeck, N. Rolim, S. Gielen, B. Pieske, E. B. Winzer, A. Linke, and V. Adams, ESC heart failure **8**, 1 (2021).

S9. R. K. Cheng, M. Cox, M. L. Neely, P. A. Heidenreich, D. L. Bhatt, Z. J. Eapen, A. F. Hernandez, J. Butler, C. W. Yancy, and G. C. Fonarow, American heart journal **168**, 5 (2014).

S10. G. C. Fonarow, W. G. Stough, W. T. Abraham, N. M. Albert, M. Gheorghiade, B. H. Greenberg, C. M. O'Connor, J. L. Sun, C. W. Yancy, and J. B. Young, Journal of the American College of Cardiology **50**, 8 (2007).

S11. J. Fielitz, M.-S. Kim, J. M. Shelton, S. Latif, J. A. Spencer, D. J. Glass, J. A. Richardson, R. Bassel-Duby, and E. N. Olson, The Journal of clinical investigation **117**, 9 (2007)
